# Environmental memory gained from exposure to extreme pCO_2_ variability promotes coral cellular acid–base homeostasis

**DOI:** 10.1098/rspb.2022.0941

**Published:** 2022-09-14

**Authors:** Kristen T. Brown, Matheus A. Mello-Athayde, Eugenia M. Sampayo, Aaron Chai, Sophie Dove, Katie L. Barott

**Affiliations:** ^1^ Department of Biology, University of Pennsylvania, Philadelphia, PA 19104, USA; ^2^ ARC Centre of Excellence for Coral Reef Studies and School of Biological Sciences, The University of Queensland, St. Lucia, Queensland 4072, Australia

**Keywords:** climate change, ocean acidification, acclimatization, environmental variability, extreme environments, coral reefs

## Abstract

Ocean acidification is a growing threat to coral growth and the accretion of coral reef ecosystems. Corals inhabiting environments that already endure extreme diel pCO_2_ fluctuations, however, may represent acidification-resilient populations capable of persisting on future reefs. Here, we examined the impact of pCO_2_ variability on the reef-building coral *Pocillopora damicornis* originating from reefs with contrasting environmental histories (variable reef flat versus stable reef slope) following reciprocal exposure to stable (218 ± 9) or variable (911 ± 31) diel pCO_2_ amplitude (μtam) in aquaria over eight weeks. Endosymbiont density, photosynthesis and net calcification rates differed between origins but not treatment, whereas primary calcification (extension) was affected by both origin and acclimatization to novel pCO_2_ conditions. At the cellular level, corals from the variable reef flat exhibited less intracellular pH (pHi) acidosis and faster pHi recovery rates in response to experimental acidification stress (pH 7.40) than corals originating from the stable reef slope, suggesting environmental memory gained from lifelong exposure to pCO_2_ variability led to an improved ability to regulate acid–base homeostasis. These results highlight the role of cellular processes in maintaining acidification resilience and suggest that prior exposure to pCO_2_ variability may promote more acidification-resilient coral populations in a changing climate.

## 1. Background

The combined impacts of ocean warming and acidification are existential threats to the structure and function of coral reef ecosystems. The increasing frequency and intensity of climate-driven marine heatwaves has provided powerful visual evidence of our changing climate, manifested by mass coral bleaching events and subsequent coral mortality [1]. The impacts of ocean acidification on reef-building corals and other reef fauna and flora are not as visually striking, though they are occurring in response to progressive increases in seawater pCO_2_ and declines in pH observed across coastal reef systems [2,3]. It is difficult to disentangle multiple co-occurring stressors and individually quantify acidification-specific effects, which often require microscopic or physico-chemical techniques to accurately examine [4–6]. However, manipulative experiments provide strong evidence that the synergistic impacts of ocean warming and acidification will lead to the dramatic decline of coral reef ecosystems by mid-to-late century if our current rate of greenhouse gas emissions are not reduced [7,8]. As the effects of climate change intensify, the growing threat of ocean acidification to reef accretion and maintenance cannot be ignored.

Naturally variable habitats provide a glimmer of hope that climate-resilient coral populations already exist on coral reefs worldwide. Variable habitats or extreme environments, such as tidally dominated back reefs and reef flats, expose organisms to short-term fluctuations in temperature and pH conditions similar to those projected for future reefs [6,9–12]. There is evidence that exposure to high diel temperature variability can promote resilience to temperature stress via holobiont adaptation or non-genetic mechanisms such as acclimatization through ‘environmental memory’, stress-tolerant endosymbiont communities (Symbiodiniaceae) and/or changes in gene expression [13–15]. The effects of pH variability on coral environmental memory, however, are less clear. Some studies have shown neutral or positive effects of pH variability on corals and other calcifiers, whereas other studies have shown negative effects, with observed differences probably a result of the magnitude of pH amplitude, the duration of the experiment and/or the response variables examined in addition to regional- and species-specific responses [6,9,10,16,17]. Further, most experiments did not characterize the long-term pH or pCO_2_ conditions from which the study specimens were collected, which can fluctuate dramatically on diurnal, seasonal and spatial scales [2,6,18]. As a result, the question remains on whether lifelong exposure to pCO_2_ variability promotes increased tolerance to acidification stress.

**Figure 1.**
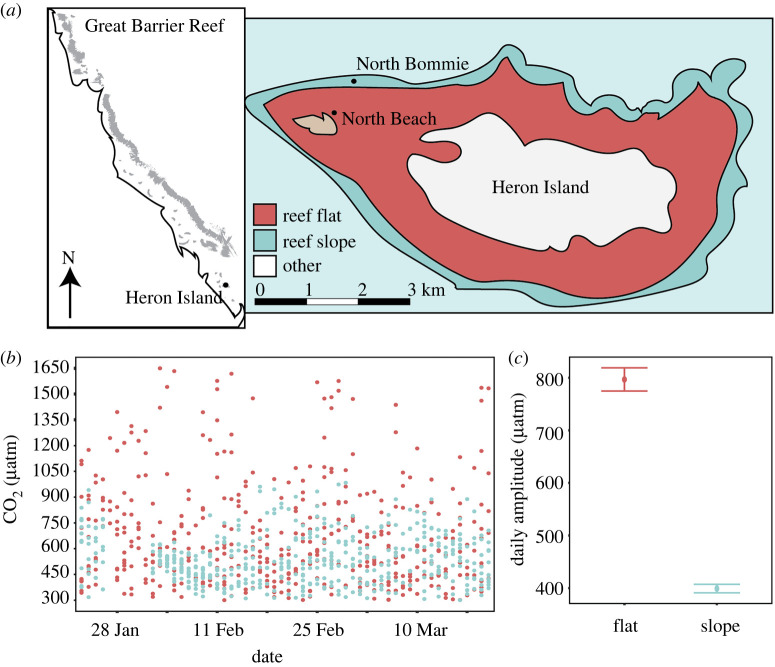
(*a*) Cartoon map of the Great Barrier Reef showing the approximate location of Heron Island, with the inset displaying the geomorphological zones and study sites at Heron Island. (*b*) *In situ* CO_2_ profiles, where points indicate individual measurements, were recorded from the reef flat and reef slope in 2016. (*c*) Daily *in situ* CO_2_ amplitude is shown as means ± s.e. (Online version in colour.)

This study examines a suite of physiological parameters to better understand how resistance to natural pH variability influences resilience to ocean acidification in a common hermatypic coral. We characterize the *in situ* pCO_2_ conditions within two habitats, a tidally dominated reef flat and an oceanic reef slope of Heron Island, southern Great Barrier Reef (figure 1), and reciprocally exposed corals of the species *Pocillopora damicornis* to replicated pCO_2_ variability from each habitat while controlling for many other factors that covary *in situ*. We investigate whether environmental memory gained from lifelong exposure to pCO_2_ variability in the field promotes a higher tolerance to acute acidification stress and if *P. damicornis* can gain or lose resilience (e.g. resistance to stress) when exposed to changed pCO_2_ regimes over a period of two months. Further, we explore the cellular mechanisms involved in coral acid–base homeostasis, how these differ between corals with distinct environmental histories, and if energetic costs are involved in driving these differences.

## 2. Methods

### (a) Study location and environmental conditions

The experiment was performed during the austral summer from mid-January to late March 2021 at Heron Island Research Station (HIRS), southern Great Barrier Reef (23 27°S, 151 55°E). Heron Reef is composed of five distinct geomorphological habitats characterized by diverse benthic communities and biogeochemical conditions [19,20]. This study focused on two distinct habitats, the reef flat (North Beach) and reef slope (North Bommie) (figure 1). Semidiurnal tidal fluctuations on the reef flat result in higher variability in temperature and CO_2_ compared to reef slope habitats, and exposes reef flat corals to extreme temperature and CO_2_ conditions projected for future reefs [11,12,20] (figure 1; electronic supplementary material, figure S1). In-field measurements (temperature, photosynthetically active radiation (PAR) and nutrients) were recorded concurrently with the manipulative experiment at the same locations where corals were collected (8 January to 18 March 2021), whereas pCO_2_ was recorded over the same season, but in 2016 (8 January to 18 March 2016; electronic supplementary material, Methods). Long-term studies show remarkable consistency in pCO_2_ measurements recorded at the same location between years [2], suggesting pCO_2_ variability measured within these identical reef habitats over the same time period may be similar across years.

### (b) Sample collection, species identification and experimental design

Fragments of the coral *P. damicornis* were collected from the reef flat and slope locations within the same depth range (1–3 m) on 14 and 15 January 2021 (figure 1). Four fragments were collected from each individual colony (genetic clones), totalling 96 fragments from 24 colonies (*n* = 12 per habitat) (electronic supplementary material, figure S2). One additional chip per colony was preserved in 100% ethanol and kept at −80°C for genetic analyses to confirm the collected coral specimens were all *P. damicornis* based on the mitochondrial ORF (cf. [21]) and identify the species of resident intracellular Symbiodiniaceae using the ITS2 rDNA and chloroplast minicircle psbA non-coding region (cf. [22,23]; full details in electronic supplementary material, Methods). All 96 collected coral fragments were standardized to a length of approximately 5 cm using bone cutters and randomly suspended with nylon fishing line from a bamboo stick (electronic supplementary material, figure S2). Six fragments were suspended from each stick and two sticks placed in each experimental treatment tank (33 l; *n* = 12 fragments per tank). To minimize ‘tank effects’, the eight tanks were randomized across one outdoor table (*n* = 4 per treatment), with each set of coral fragments rotated into an adjacent tank of the same treatment every third day. Tanks and lids were covered with filters (Old Steel Blue no. 725, Lee Filters) to mimic the light environment at the collection sites (figure 1; electronic supplementary material, figures S1 and S3). Notable paling was observed during the first week of the experiment, so light intensities were reduced with an additional shade cloth (electronic supplementary material, figure S1). All surfaces including exposed cut coral bases were cleaned every 3 days to remove any epilithic algae.

**Figure 2.**
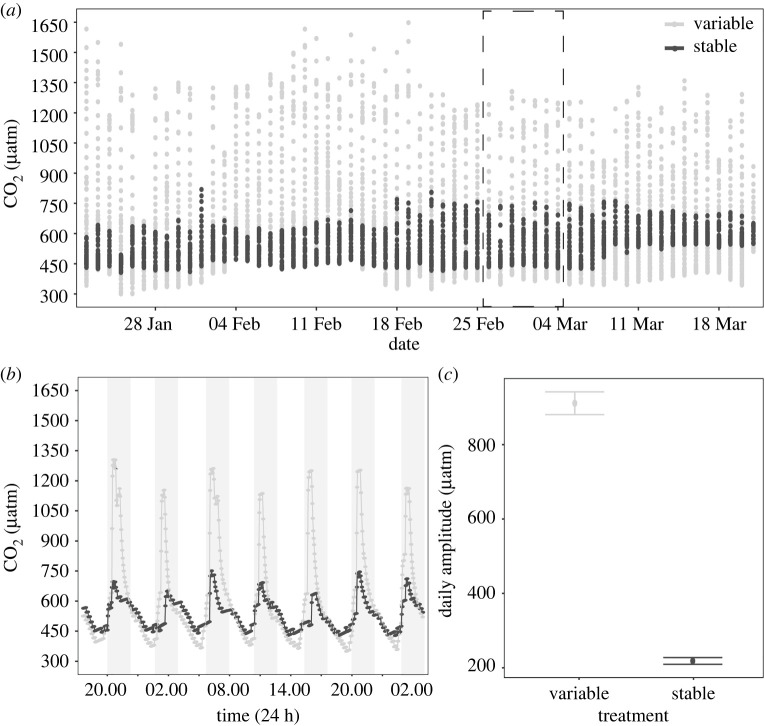
(*a*) Upstream CO_2_ conditions measured in experimental sumps over the course of the experiment. Points indicate individual measurements and the dashed box highlights the subset represented in (*b*). (*b*) A one week subset of CO_2_ conditions demonstrating diel variability. Points indicate individual measurements and grey shading indicates periods of darkness. (*c*) Daily CO_2_ amplitude is shown as means ± s.e.

After 7 days of recovery from collection and handling, corals were exposed to two distinct treatments for eight weeks: (i) stable pCO_2_ and (ii) variable pCO_2_ (figure 2), which were maintained following previously described methods [8,24] (electronic supplementary material, Methods and figure S3). Upstream CO_2_ was continuously recorded in treatment sumps (figure 2) and within experimental tanks, seawater temperature (HOBO pendant logger) and PAR (Odyssey PAR sensor) were continuously measured at 30 min intervals in each treatment by randomly rotating two probes per treatment between tanks (electronic supplementary material, figure S1). Weekly samples (*n* = 3 per treatment) were collected for total alkalinity (*A*_T_) and pH_Total_ at midday and midnight. *A*_T_ was determined via the Gran titration method using 0.1 M HCl and pH_Total_ was determined via a high-precision glass pH electrode (DGi101-SC, Mettler Toledo) across replicated 20 g seawater samples [25]. Acid concentration was calibrated at the beginning of each titration session using the certified reference materials from the Dickson Laboratory at Scripps Institute of Oceanography, USA. Salinity was measured via refractometer and remained constant at 35.0 throughout the experiment. Parameters of the seawater carbonate chemistry, including carbonate, bicarbonate, aragonite saturation state, were calculated from our temperature, salinity, *A*_T_ and pH_Total_ measurements using the *seacarb* package in R [26] (table 1).

**Table 1.** Mean carbonate chemistry throughout the two months experiment. All values are displayed as means ± s.e. (*n* = 27). pCO_2_ and aragonite saturation state (Ω_arag_) were calculated from pH_Total_, total alkalinity (*A*_T_), temperature and salinity using the package *seacarb* in R [26]. Salinity remained constant at 35.0 throughout the experiment.

time of day	treatment	temp (°C)	pCO_2_ (μatm)	*A*_T_ (μmol kg^−1^)	pH_Total_	Ω_arag_
midday	stable	27.42 ± 0.04	288.4 ± 5	2279 ± 6	8.15 ± 0.006	4.34 ± 0.05
midday	variable	27.42 ± 0.04	283.6 ± 9	2273 ± 4	8.15 ± 0.01	4.39 ± 0.08
midnight	stable	27.41 ± 0.04	368.7 ± 18	2263 ± 6	8.07 ± 0.02	3.79 ± 0.11
midnight	variable	27.41 ± 0.04	582.2 ± 27	2268 ± 5	7.91 ± 0.02	2.90 ± 0.13

### (c) Physiological analyses

Coral survivorship was assessed visually daily, and only one coral fragment died during the experiment. Net calcification, surface area (a proxy for extension [11]), volume and dark-adapted photosynthetic efficiency (*F*_v_/*F*_m_) of coral fragments were measured six times during the experiment (approx. two weeks intervals) via buoyant weight and photogrammetry using previously described methods [27–29] (electronic supplementary material, Methods, figures S4 and S5). At the end of the experiment, metabolic rates (net photosynthesis, dark respiration and light-enhanced dark respiration) were assessed via changes in oxygen evolution using oxygen optodes connected to an OXY-10 (PreSens) optical analyser [30] (electronic supplementary material, Methods). Upon completion of these living analyses, half of the coral fragments were flash frozen in liquid nitrogen and stored at −80°C. Subsequent laboratory analyses were done on these 48 specimens. For these analyses, corals (*n* = 12) were water-piked on ice to remove coral tissue from the skeleton using 50 ml of 0.1 M phosphate buffered saline solution. The tissue slurry was centrifuged at 4°C once for 5 min at 2500*g* to sufficiently separate host tissue and the intracellular endosymbiont cells. Host tissue was analysed for host-soluble protein concentration and mycosporine-like amino acids (MAAs) concentrations spectrophotometrically [31]. Endosymbiont densities were determined from cell counts of three aliquots using a haemocytometer [30]. Host protein concentration and endosymbiont cell densities were standardized to surface area (cm^2^), which was determined using the single wax-dipping technique [32], whereas MAAs were normalized to host protein content. Endosymbiont photopigments were extracted in 100% acetone for 24 h and concentration of chlorophyll *a* were determined via absorbance at 630, 663 and 750 nm using the equations in [33]. Pigment concentrations were standardized to both surface area and endosymbiont densities. Wax-dipping was also used to determine calcium carbonate (CaCO_3_) bulk density, where the skeleton was sealed with a coat of wax, dry weighed and then buoyant weighed [34]. The difference between dry weight and buoyant weight was calculated to determine the bulk volume, which was subtracted from the dry weight to yield bulk density. The other half of the fragments were transported alive from Heron Island to the University of Queensland, Brisbane to assess intracellular acid–base status and acidification resilience following established methods [35]. Briefly, cells were loaded with SNARF-1AM and imaged using a confocal microscope (Zeiss LSM 710) via excitation at 561 nm, with SNARF-1 fluorescence emission acquired in two channels (585 and 640 ± 10 nm) simultaneously (see full details in electronic supplementary material, Methods).

### (d) Statistical analysis

Seawater temperature, CO_2_, PAR and nutrient concentrations were analysed for differences within and between experiment treatments (treatment: stable, variable) and reef habitats (origin: reef flat, reef slope). The effect of tide (tide: high, low) was also explored on nutrient concentrations. The interactive effects of origin and treatment were explored on *P. damicornis* growth (net calcification, extension, CaCO_3_ density) and physiology (dark respiration, light-enhanced dark respiration, host-soluble protein, MAAs, net photosynthesis, photosynthetic efficiency, endosymbiont density, chlorophyll *a* concentration) using linear mixed effects (lme) models with colony genotype as a random effect [36]. Similarly, the interactive effects of origin, treatment and cell type (i.e. cells lacking microalgal endosymbionts (non-symbiocytes) and containing microalgal endosymbionts (symbiocytes)) were explored on intracellular pH (pHi) using a lme model. Colony genotype was included as a random effect in all lme models. All data met assumptions (homogeneity of variance, normality of distribution) through graphical analyses of residual plots. The significance of fixed effects and their interactions was determined using an analysis of variance with a type III error structure using the Anova function in the *car* package [37]. Significant interactive effects were followed by pairwise comparison of estimate marginal means using the *emmeans* package with Tukey HSD adjusted *p-*values [38]. All statistical analyses were done using R v. 4.0.0 software [39], and graphical representations were produced using the package *ggplot2* [40].

## 3. Results and discussion

### (a) Physico-chemical conditions differed between habitats and treatments

The mean seawater pCO_2_ (µatm) *in situ* (measured in 2016) was similar between the reef flat (454 ± 3.0) and reef slope (418 ± 1.9), but the reef flat experienced twice the mean daily pCO_2_ amplitude than the reef slope (797 ± 20 versus 399 ± 8 d^−1^, respectively; figure 1). A number of other environmental conditions covaried with pCO_2_ between the two habitats, including temperature and PAR. While the mean temperature (°C) was the same between the reef flat (26.8 ± 0.02) and reef slope (26.8 ± 0.01), the daily amplitude was nearly three times greater on the reef flat than the reef slope (4.3°C versus 1.3°C; electronic supplementary material, figure S1). Mean PAR (µmol quanta m^−2^ s^−1^) was significantly higher on the reef flat than the reef slope (456 ± 7.2 versus 288 ± 4.5; *F* = 2391, *p* < 0.0001; electronic supplementary material, figure S1), driven by tidal fluctuations in depth. Seawater nutrient concentrations (ammonium, nitrate, nitrite, phosphate) were not significantly different between habitats (*F* = 0.56, *p* = 0.57) or between low or high tide (*F* = 0.16, *p* = 0.69) throughout the experimental period (electronic supplementary material, figure S6).

Within the experimental treatments, seawater pCO_2_ dynamics differed significantly between treatments. Specifically, there was a 4.2-fold difference in mean diel pCO_2_ amplitude (µatm) between the variable (911.3 ± 30.69) and stable (218.3 ± 9.14) treatments across the experimental period in the upstream sumps, which resulted in a mean pCO_2_ that was slightly higher in the variable treatment (620 ± 3.4) compared to the stable treatment (540 ± 0.9) (*F* = 506, *p* < 0.0001; figure 2). Downstream of the sumps, the physiological activity of the corals influenced pCO_2_ within the experimental tanks, resulting in a lower mean pCO_2_ (µatm) conditions in the stable (327.8 ± 10.5) and variable (435.7 ± 25.3) treatments, which were more similar to mean pCO_2_ conditions measured *in situ*. Importantly, tank pCO_2_ was significantly influenced by the interaction between treatment and time (*F* = 40.8, *p* < 0.0001), with pairwise comparisons revealing no difference in pCO_2_ conditions during midday (*p* = 0.99) but significantly higher pCO_2_ in the variable treatment during the programmed spike in conditions at midnight (*p* < 0.0001; table 1). Similarly, pH within experimental tanks was significantly influenced by the interaction between treatment and time (*F* = 33.7, *p* < 0.0001). At midday, however, there was no difference in mean pH between treatments (8.15) (*p* = 0.98; table 1), while pairwise comparisons showed pH in the variable treatment was approximately 0.15 pH units lower than the stable treatment at the midnight peak in pCO_2_ (*p* < 0.0001; table 1). As expected due to controlled tank conditions, temperature, irradiance and nutrients did not differ within and across experimental treatments (electronic supplementary material, figures S1 andS6). The mean temperature was 27.4°C, below Heron Island's bleaching threshold (maximum monthly mean (MMM) +1°C of 28.3°C), and mean PAR was approximately 125 µmol quanta m^2^ s^−1^ throughout the experiment. Generally, these experimental conditions were more similar to the reef slope than flat conditions (electronic supplementary material, figure S1). This successful maintenance of consistent temperature, PAR and nutrient concentrations between treatments suggests that differences in pCO_2_ amplitude throughout the experiment are likely drivers of the physiological responses observed.

### (b) Consistency in coral and Symbiodiniaceae species between habitats suggests important role of organismal acclimatization at local scale

All coral colonies used in the experiments were confirmed to be *P. damicornis* (GenBank accession numbers OP296503–OP296521; 100% match to Pocillopora type alpha cf. [21] with GenBank accession numbers JX985598 and JX985606). These results align with earlier studies, which also exclusively found *P. damicornis* across the reef flat and reef slope environment at Heron Reef [41]. The microalgal endosymbionts were all consistent with ITS2-type C1-b-c/C42-a (cf. [22]), containing co-dominant rDNA repeats identified as sequence alpha-numericals C1, C1b, C1c, C42, C42a present in characteristic ‘fingerprint’ DGGE profiles of each sample. The ITS2 rDNA data, coupled with the phylogenetic analyses of psbA sequences (electronic supplementary material, figure S7; GenBank accession numbers OP279755–OP279774), support that all coral specimens contained the recently described pocilloporid-specific endosymbiont *Cladocopium latusorum* [42]. Our findings that corals from the different habitats were indeed the same species for both the host and endosymbiont are important to our understanding of the effects of pCO_2_ variability on coral physiology, as it reduces confounding species-specific effects that often covary with habitat. Earlier studies at the study location have demonstrated divergent Symbiodiniaceae species associated with *P. damicornis* inhabiting the reef flat (*C. latusorum*; previously type ‘C42a’) or slope (*Cladocopium* type ‘C33a’) [22,41,43]. Surprisingly, none of the samples in the present study were found to contain *Cladocopium* ‘C33a’. The discrepancy with prior studies may be due to: (i) depth, with reef slope *P. damicornis* sampled at shallower depths (1–3 m) in this study relative to others (>4 m) [22,41,43]; (ii) spatial variability, with reef slope *P. damicornis* sampled on the open-ocean side of Heron Reef, in contrast to the turbid channel between Wistari and Heron Reef sampled by others (figure 1) [22,41,43]; and/or (iii) thermal stress, as our study was conducted 1 year after significant heat stress and coral bleaching occurred [44]. In general, *Cladocopium* type ‘C33a’ is more specialized to deeper, darker, or turbid areas [43] and may have been more sensitive to the 2020 bleaching (approx. nine months prior), whereas *C. latusorum* appears to be more of a generalist [42] and may have been less affected, ensuring its retention. It has been suggested that adaptation to the harsh environmental conditions of the reef flat may, in part, stem from an association with divergent Symbiodiniaceae species [41]. Although all endosymbionts were identified as *C. latusorum*, the psbA region indicates substantial genotypic diversity in this group but based on our study samples no identifiably consistent differences were observed between habitats (electronic supplementary material, figure S7). Albeit based on a small sample size, the consistency of Symbiodiniaceae species and genotypic diversity between slope and flat habitats suggests that acclimatization plays a significant role in tolerance to environmental variability. Indeed, holobiont adaptation in response to distinct environmental regimes can occur in the absence of distinct endosymbiont genotypes [13], and, even in host–symbiont specific associations, such as those belonging to the family Pocilloporidae, a large degree of phenotypic plasticity to varying environmental conditions at small spatial scales has been observed [45,46].

### (c) Environmental memory of native physico-chemical conditions drove phenotypic plasticity

To cope with living under the stress imposed by variable or extreme environments, corals exhibit remarkable phenotypic plasticity [47,48]. The ability of corals to change their physiological performance in response to the environment has, however, been linked to trade-offs between different physiological traits [13]. Here, corals predominantly maintained similar physiological activity to conspecifics from their native environment, regardless of being grown under stable or variable pCO_2_ for two months. For example, symbiont density was significantly influenced by origin (*F* = 6.18, *p* = 0.013), with corals originating from the reef flat containing lower symbiont densities than corals from the slope. This could be a carry-over effect of sublethal stress from the harsh temperature and light regimes of the reef flat habitat (electronic supplementary material, figure S1). Interestingly, net photosynthesis was slightly higher in corals originating from the reef flat regardless of treatment (origin: *F* = 4.15, *p* = 0.042) (electronic supplementary material, figure S8), whereas dark respiration (*F* = 0.007, *p* = 0.9), light-enhanced dark respiration (*F* = 0.23, *p* = 0.63) and photosynthesis to respiration (P : R) ratios (*F* = 0.03, *p* = 0.85) displayed no significant patterns between origin or treatment (electronic supplementary material, figure S9). These observations suggest that autotrophic energy acquisition was not impeded by the daily, extreme, oscillations in pCO_2_ to which these corals were acclimatized. This is in contrast to corals exposed to simulated seawater acidification and/or *in situ* thermal stress, which typically exhibit reductions in metabolic rates [30,35]. In addition, there were no significant differences in chlorophyll *a* concentrations (*F* = 0.21, *p* = 0.65) or photochemical efficiency (*F*_v_/*F*_m_) between origin (*F* = 1.25, *p* = 0.26) or treatment (*F* = 0.01, *p* = 0.91) (electronic supplementary material, figure S8), suggesting that pCO_2_ variability did not have an effect on these parameters. Finally, host protein content, a proxy for biomass, showed no significant differences between origin or treatment (*F* = 0, *p* = 0.99) (electronic supplementary material, figure S9), in contrast to others that have found corals originating from variable habitats tend to have higher tissue biomass than conspecifics from stable habitats due to a prioritization of biomass over calcification in harsher environments [9,13].

**Figure 3.**
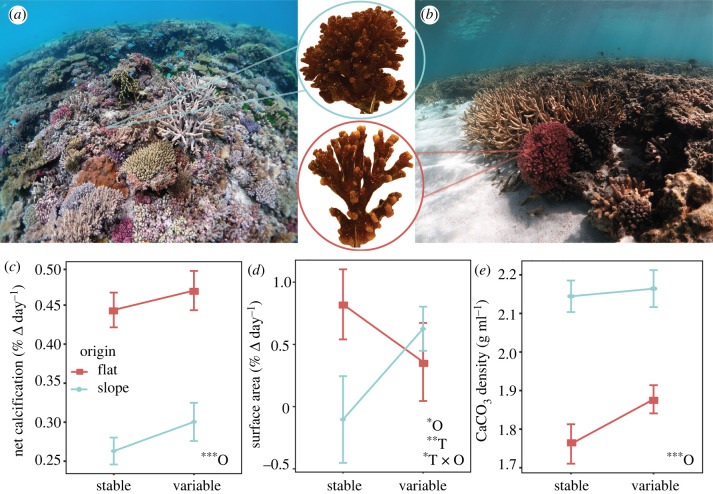
(*a*) Representative images of the reef slope at North Bommie and (*b*) the reef flat at North Beach, Heron Island, southern Great Barrier Reef. Insets show representative *Pocillopora damicornis* fragments at the end of the two months experiment, demonstrating analogous phenotypic behaviour to the habitat of origin. (*c*) Net calcification rates, (*d*) surface area and (*e*) calcium carbonate (CaCO_3_) density by treatment and origin, displayed as means ± s.e. Insets indicate statistical significance (*p < 0.05, **p < 0.001, ***p < 0.0001) of individual and interactive effects for origin (O) and treatment (T) as determined from linear mixed effects models.

Coral net calcification (%ΔBW day^−1^) was significantly affected by origin, with higher rates in *P. damicornis* that originated from the reef flat (*F* = 7.24, *p* = 0.007), while the effect of treatment was not significant (*F* = 1.15, *p* = 0.28) (figure 3). However, when examining the individual effects of primary calcification (i.e. extension) and secondary calcification (i.e. densification) separately, treatment emerged as a significant factor. For example, surface area (%ΔSA day^−1^), a proxy for extension, showed a significant interaction between treatment and origin (*F* = 5.82, *p* = 0.015). Specifically, corals from the reef flat increased their extension rates in the stable (non- native) treatment, which could indicate a release from stressful pCO_2_ conditions (figure 3). Secondary calcification, measured as CaCO_3_ bulk density, was found to be lower in *P. damicornis* originating from the reef flat compared to the slope (*F* = 37.0, *p* < 0.0001), but treatment had no effect (figure 3). Lower skeletal density, coupled with higher net calcification rates, indicates that corals from the reef flat were extending at higher rates than corals from the reef slope, resulting in longer branches with more porous skeletons (figure 3). Given the amount of new growth observed (approx. 15–30% of fragment), however, we were unable to capture changes in density due to treatment (i.e. acclimatization to novel experimental pCO_2_ conditions); however, as net calcification did not change while extension increased in response to novel pCO_2_ conditions, it is likely that densification correspondingly decreased. Future studies could isolate the new growth area to better resolve morphological changes (e.g. diameter of calyx, porosity) resultant from acclimatization to pCO_2_ variability. The origin-specific differences in CaCO_3_ density that we observed aligns with habitat-specific patterns observed in earlier studies, where skeletal density was a direct reflection of wave exposure or water motion (e.g. lower CaCO_3_ density in protected reef flats or lagoons compared to conspecifics from high wave energy reef slopes) [11,48], but lower densification can also occur in response to high pCO_2_ [11,34]. Indeed, strong morphological variation and branch modularity was also qualitatively observed, with *P. damicornis* native to the reef flat continuing to exhibit lower density of branches and greater branch thickness than corals native to the reef slope (figure 3). Similar macro-morphological patterns in pocilloporids have been attributed to flow dynamics [48] and even storm frequency [47]. It can take as long as six months of exposure to a new environment to induce gross morphological changes in *P. damicornis* [47], and thus more time may have been needed for organisms to fully converge on the same phenotypes. Future experiments could, therefore, be expanded over longer time frames to tease apart the influence of lifelong acclimatization versus adaptation to diel pCO_2_ variability on the different aspects of coral calcification.

### (d) Improved ability to control acid–base homeostasis indicates acclimatization to extreme diel pCO_2_ oscillations

**Figure 4.**
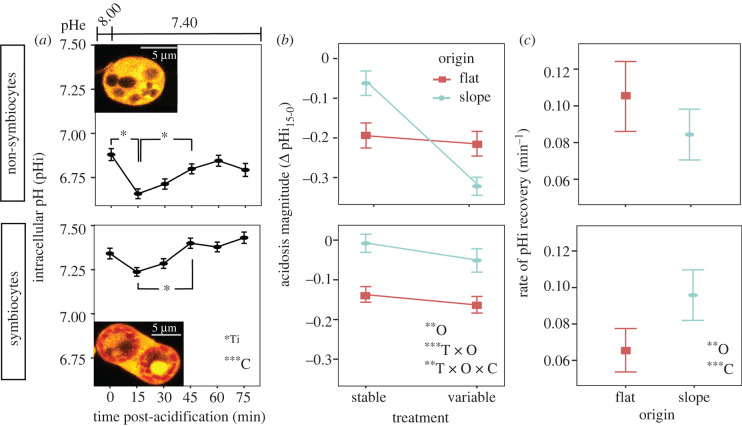
(*a*) Intracellular pH (pHi) of cells from *Pocillopora damicornis* corals following exposure to acidified seawater (pHe 7.4) for non-symbiocytes (top) and symbiocytes (bottom) over time by cell type. Insets show the fluorescence micrograph of cells lacking microalgal endosymbionts and containing microalgal endosymbionts loaded with SNARF-1 AM (orange; endosymbiont autofluorescence is indicated in red). (*b*) The initial change in pHi in response to acidification stress (acidification magnitude; ΔpHi_15-0_) by treatment, origin and cell type. (*c*) The rate of pHi recovery (pH min^−1^) by origin and cell type. Statistical significance (*p < 0.05, **p < 0.001, ***p < 0.0001) is displayed for individual and interactive effects for time (Ti), origin (O), treatment (T) and cell type (C) as determined from linear mixed effects models. All data are displayed as means ± s.e.

Maintaining stable intracellular pH (pHi) is critical for cellular physiology [49] and requires the capacity to sense pH changes that may result from internal and external sources [50], and to regulate downstream compensatory pH pathways [51]. Similarly, biologically driven elevation of the pH and aragonite saturation state of the extracellular calcifying medium (ECM) by the surrounding calicodermis is essential for coral calcification [52–54]. Acid–base regulation of the ECM and pHi are closely linked, with pH of the ECM and pHi of *P. damicornis* both displaying a positive relationship with increasing seawater pH in both light and dark conditions [55]. Here, we tested whether acclimatization to seawater pCO_2_ variability altered coral acid–base homeostasis dynamics by exposing *P. damicornis* cells to acidification stress (pHe 7.40). Symbiocytes had higher pHi than non-symbiocytes (cell type: *F* = 42.9, *p* < 0.0001), and overall, coral cells both with and without endosymbionts exhibited an initial acidification of pHi after exposure to acidified seawater, followed by recovery to their initial pHi setpoint, indicative of an active physiological compensatory response in all cell types (time: *F* = 2.2, *p* = 0.049) (figure 4*a*). Interestingly, the initial change in pHi in response to acidification stress (i.e. acidosis magnitude after 15 min exposure; ΔpHi_15–0_) showed a significant three-way interaction between treatment, origin and cell type (*F* = 4.33, *p* = 0.003) (figure 4*b*). Pairwise comparisons revealed that in non-symbiocytes, there was no difference in acidification magnitude for *P. damicornis* originating from the reef flat and acclimated to either stable or variable pCO_2_ treatments (*p* = 0.6). This suggests corals native to the flat had a robust ability to buffer pHi and maintain acid–base homeostasis in the face of acidification stress regardless of treatment, possibly due to adaptation and/or constitutive expression of regulatory mechanisms to compensate for extreme pCO_2_ oscillations. While the specific mechanisms were not investigated in this study, it is possible that the upregulation of acid–base homeostasis mechanisms in corals native to the reef flat, such as the proteins involved in pH-sensing [50], proton or carbonate ion transport [56–59], or conversion of carbon dioxide into bicarbonate (e.g. carbonic anhydrases) [60], ultimately primed *P. damicornis* native to the reef flat to cope with acute pH stress. This behaviour of ‘front-loading’ stress response pathways has been seen in coral temperature variability responses [13], and is an important avenue for future investigation in coral acidification resilience, especially as many of these mechanisms are also likely involved in calcification [53,57]. Also, corals may decrease investment in upregulation ECM pH during times of acid stress (e.g. by inhibiting Ca^2+^-ATPase in the calicodermis, which may uptake protons from the ECM [56]), whereby cells could reduce intracellular acidosis, but this may come at the expense of calcification.

Interestingly, in contrast to corals native to the reef flat, *P. damicornis* native to the reef slope but exposed to the variable conditions showed a three-fold drop in pHi following exposure to acidified seawater relative to slope corals remaining in their native stable pCO_2_ conditions (*p* < 0.0001), and this acidification was of significantly larger magnitude than that of corals originating from the flat in either treatment (*p* = 0.002; figure 4*b*). This large drop in pHi following acute external acidification suggests that corals adapted to stable reef slope conditions have a limited ability to buffer their internal pH after eight weeks of exposure to extreme diel pCO_2_ variability. Again, the mechanisms behind this response are unknown, but are possibly due to an inability to further upregulate acid–base regulatory machinery during an acute acid challenge following chronic exposure to extreme diel pH variability and/or a lower passive buffering capacity. Furthermore, following the initial acidosis, *P. damicornis* native to the reef slope exhibited slower pHi recovery rates relative to corals native to the flat (*F* = 4.26, *p* = 0.039; figure 4*c*), suggesting that corals native to the reef slope have a less robust capacity to compensate for acidification stress than corals native to the reef flat. This investment in acid–base regulation by reef flat corals may be beneficial for coping with ocean acidification, but could also come at a cost. For example, our data appear to demonstrate a trade-off in response to extreme pCO_2_ variability, where greater investment in acid–base homeostasis leaves insufficient energy to support secondary calcification. If reef flat corals must expend more energy on pHi regulation, such as through the active (i.e. ATP-dependent) removal of protons from their tissues, this could force a trade-off with expensive energy investments such as skeletal infrastructure (e.g. organic matrix [61]; removal of protons from the calcification site [56]; transport of Ca^2+^ through the calicodermis to the ECM [62]), possibly slowing or ceasing calcification altogether. In addition, as seawater acidification leads to more acidic conditions within the gastrovascular cavity (coelenteron) [63], particularly the acidic region lining the aboral tissues [64], it becomes more energetically costly for corals to move protons from the ECM through the calicodermis and into the coelenteron. Finally, symbiocytes displayed faster pHi recovery rates than non-symbiocytes (*F* = 34.9, *p* < 0.0001; figure 4*c*), suggesting that endosymbionts play a significant role in helping buffer host cells following acidification stress. The mechanisms remain unknown, but could occur through energetic provisioning to the host that supports the energetic demands of acid–base homeostasis (e.g. ATPase activity; intracellular trafficking of ion channels), or possibly through their own metabolic activity [5]. However, as our experiments were conducted in the dark, this mechanism is not due to CO_2_ consumption via photosynthesis, as can occur in the light [5]. Despite the encouraging signs that these corals living in extreme pCO_2_ conditions are able to better cope with acute acidification stress, as climate change intensifies, the interactive effects of ocean warming and acidification could interact to undermine the ability of corals to regulate acid–base homeostasis [35], ultimately weakening CaCO_3_ structures that support entire reef ecosystems and coastal communities [7,8].

## 4. Conclusion

Our results highlight that pCO_2_ oscillations, in addition to commonly recognized parameters like temperature, water motion and light, play an important role in influencing phenotypic variability in calcification between extreme environments and suggest that acclimatization to pCO_2_ variability may promote acidification-resilient populations in the future. However, energy investments into regulating acid–base homeostasis will become more costly in a more acidic ocean [60] and tolerance to one stressor may come at a cost to others (e.g. storms, marine heatwaves). Furthermore, as ocean warming and acidification intensify, corals will be pushed to the edge of their physiological limits, with tolerance to present-day variability not necessarily conferring resilience to future ocean warming and acidification [9,11]. While more research is needed to determine how long it takes to acquire resistance to acidification stress, it is evident from this study that *P. damicornis* native to stable environments cannot acclimatize to extreme pCO_2_ oscillations conditions over relatively short time scales. Nevertheless, our results suggest that reef corals may be more resistant to future ocean acidification conditions in extreme environments where diel variation in seawater pCO_2_ is prominent, which has important implications for reef persistence in a changing climate.

## Data Availability

The datasets and scripts generated/analysed for this study as well as the sequence files (fasta format) can be found as an electronic notebook on https://github.com/imkristenbrown/pCO2-variability-promotes-coral-cellular-acid-base-homeostasis. The data are provided in electronic supplementary material [65].

## References

[1] Hughes TP et al. 2017 Global warming and recurrent mass bleaching of corals. Nature **543**, 373-377. (10.1038/nature21707) 28300113

[2] Fabricius KE, Neill C, Van Ooijen E, Smith JN, Tilbrook B. 2020 Progressive seawater acidification on the Great Barrier Reef continental shelf. Sci. Rep. **10**, 18602. (10.1038/s41598-020-75293-1) 33110129 PMC7592051

[3] Mongin M et al. 2016 The exposure of the Great Barrier Reef to ocean acidification. Nat. Commun. **7**, 10732. (10.1038/ncomms10732) 26907171 PMC4766391

[4] Venn AA, Tambutte E, Lotto S, Zoccola D, Allemand D, Tambutte S. 2009 Imaging intracellular pH in a reef coral and symbiotic anemone. Proc. Natl Acad. Sci. USA **106**, 16 574-16 579. (10.1073/pnas.0902894106) PMC2757848 19720994

[5] Gibbin EM, Putnam HM, Davy SK, Gates RD. 2014 Intracellular pH and its response to CO_2_-driven seawater acidification in symbiotic versus non-symbiotic coral cells. J. Exp. Biol. **217**, 1953-1969. (10.1242/jeb.099549) 24625648

[6] Cornwall CE, Comeau S, DeCarlo TM, Moore B, D'Alexis Q, McCulloch MT. 2018 Resistance of corals and coralline algae to ocean acidification: physiological control of calcification under natural pH variability. Proc. R. Soc. B **285**, 20181168. (10.1098/rspb.2018.1168) PMC6111182 30089625

[7] Eyre BD, Cyronak T, Drupp P, De Carlo EH, Sachs JP, Andersson AJ. 2018 Coral reefs will transition to net dissolving before end of century. Science **359**, 908-911. (10.1126/science.aao1118) 29472482

[8] Dove SG, Brown KT, Van Den Heuvel A, Chai A, Hoegh-Guldberg O. 2020 Ocean warming and acidification uncouple calcification from calcifier biomass which accelerates coral reef decline. Commun. Earth Environ. **1**, 1-9. (10.1038/s43247-020-0001-2)

[9] Camp EF, Smith DJ, Evenhuis C, Enochs I, Manzello D, Woodcock S, Suggett DJ. 2016 Acclimatization to high-variance habitats does not enhance physiological tolerance of two key Caribbean corals to future temperature and pH. Proc. R. Soc. B **283**, 20160442. (10.1098/rspb.2016.0442) PMC4892798 27194698

[10] Rivest EB, Comeau S, Cornwall CE. 2017 The role of natural variability in shaping the response of coral reef organisms to climate change. Curr. Clim. Change Rep. **3**, 271-281. (10.1007/s40641-017-0082-x)

[11] Rathbone M, Brown KT, Dove S. 2021 Tolerance to a highly variable environment does not infer resilience to future ocean warming and acidification in a branching coral. Limnol. Oceanogr. **67**, 272-284. (10.1002/lno.11991)

[12] Camp EF, Schoepf V, Mumby PJ, Hardtke LA, Rodolfo-Metalpa R, Smith DJ, Suggett DJ. 2018 The future of coral reefs subject to rapid climate change: lessons from natural extreme environments. Front. Mar. Sci. **5**, 4. (10.3389/fmars.2018.00004)

[13] Kenkel CD, Matz MV. 2016 Gene expression plasticity as a mechanism of coral adaptation to a variable environment. Nat. Ecol. Evol. **1**, 14. (10.1038/s41559-016-0014) 28812568

[14] Oliver TA, Palumbi SR. 2011 Do fluctuating temperature environments elevate coral thermal tolerance? Coral Reefs **30**, 429-440. (10.1007/s00338-011-0721-y)

[15] Brown KT, Barott KL. 2022 The costs and benefits of environmental memory for reef-building corals coping with recurring marine heatwaves. Integr. Comp. Biol. **6**, icac074. (10.1093/icb/icac074) 35661887

[16] Chan WY, Eggins SM. 2017 Calcification responses to diurnal variation in seawater carbonate chemistry by the coral *Acropora formosa*. Coral Reefs **36**, 763-772. (10.1007/s00338-017-1567-8)

[17] Comeau S, Edmunds PJ, Spindel NB, Carpenter RC. 2014 Diel pCO_2_ oscillations modulate the response of the coral *Acropora hyacinthus* to ocean acidification. Mar. Ecol. Prog. Ser. **501**, 99-111. (10.3354/meps10690)

[18] Cyronak T et al. 2020 Diel temperature and pH variability scale with depth across diverse coral reef habitats. Limnol. Oceanogr. Lett. **5**, 193-203. (10.1002/lol2.10129)

[19] Phinn SR, Roelfsema CM, Mumby PJ. 2012 Multi-scale, object-based image analysis for mapping geomorphic and ecological zones on coral reefs. Int. J. Remote Sens. **33**, 3768-3797. (10.1080/01431161.2011.633122)

[20] Brown KT, Bender-Champ D, Kubicek A, van der Zande R, Achlatis M, Hoegh-Guldberg O, Dove SG. 2018 The dynamics of coral-algal interactions in space and time on the southern Great Barrier Reef. Front. Mar. Sci. **5**, 181. (10.3389/fmars.2018.00181)

[21] Schmidt-Roach S, Lundgren P, Miller KJ, Gerlach G. 2013 Assessing hidden species diversity in the coral *Pocillopora damicornis* from Eastern Australia. Coral Reefs **32**, 161-172.

[22] Sampayo EM, Dove S, LaJeunesse TC. 2009 Cohesive molecular genetic data delineate species diversity in the dinoflagellate genus Symbiodinium. Mol. Ecol. **18**, 500-519. (10.1111/j.1365-294X.2008.04037.x) 19161470

[23] LaJeunesse TC, Thornhill DJ. 2011 Improved resolution of reef-coral endosymbiont (Symbiodinium) species diversity, ecology, and evolution through psbA non-coding region genotyping. PLoS ONE **6**, e29013. (10.1371/journal.pone.0029013) 22216157 PMC3247227

[24] Dove SG, Kline DI, Pantos O, Angly FE, Tyson GW, Hoegh-Guldberg O. 2013 Future reef decalcification under a business-as-usual CO_2_ emission scenario. Proc. Natl Acad. Sci. USA **110**, 15 342-15 347. (10.1073/pnas.1302701110) PMC3780867 24003127

[25] Kline DI et al. 2012 A short-term in situ CO_2_ enrichment experiment on Heron Island (GBR). Sci. Rep. **2**, 413. (10.1038/srep00413) 22639723 PMC3356889

[26] Gattuso J-P et al. 2021 Package ‘seacarb’. See https://jpgattuso.github.io/seacarb.html.

[27] Davies PS. 1989 Short-term growth measurements of corals using an accurate buoyant weighing technique. Mar. Biol. **101**, 389-395. (10.1007/BF00428135)

[28] Brown KT et al. 2021 Habitat-specific biogenic production and erosion influences net framework and sediment coral reef carbonate budgets. Limnol. Oceanogr. **66**, 349-365. (10.1002/lno.11609)

[29] Ferrari R, McKinnon D, He H, Smith R, Corke P, González-Rivero M, Mumby P, Upcroft B. 2016 Quantifying multiscale habitat structural complexity: a cost-effective framework for underwater 3D modelling. Remote Sens. **8**, 113. (10.3390/rs8020113)

[30] Brown KT, Bender-Champ D, Kenyon TM, Rémond C, Hoegh-Guldberg O, Dove S. 2019 Temporal effects of ocean warming and acidification on coral–algal competition. Coral Reefs **38**, 297-309. (10.1007/s00338-019-01775-y)

[31] Whitaker JR, Granum PE. 1980 An absolute method for protein determination based on difference in absorbance at 235 and 280 nm. Anal. Biochem. **109**, 156-159. (10.1016/0003-2697(80)90024-X) 7469012

[32] Holmes G. 2008 Estimating three-dimensional surface areas on coral reefs. J. Exp. Mar. Biol. Ecol. **365**, 67-73. (10.1016/j.jembe.2008.07.045)

[33] Jeffrey SW, Humphrey GF. 1975 New spectrophotometric equations for determining chlorophylls a, b, c1 and c2 in.higher plants, algae and natural phytoplankton. Biochem. Physiol. Pflanz. **167**, 191-194. (10.1016/S0015-3796(17)30778-3)

[34] Tambutté E, Venn AA, Holcomb M, Segonds N, Techer N, Zoccola D, Allemand D, Tambutté S. 2015 Morphological plasticity of the coral skeleton under CO_2_-driven seawater acidification. Nat. Commun. **6**, 1-9. (10.1038/ncomms8368) PMC4490415 26067341

[35] Innis T et al. 2021 Marine heatwaves depress metabolic activity and impair cellular acid–base homeostasis in reef-building corals regardless of bleaching susceptibility. Glob. Change Biol. **27**, 2728-2743. 10.1111/gcb.15622 33784420

[36] Bates D, Mächler M, Bolker B, Walker S. 2015 Fitting linear mixed-effects models using lme4. J. Stat. Softw. **67**, 1-48. (10.18637/jss.v067.i01)

[37] Fox J et al. 2012 Package ‘car’. See https://cran.r-project.org/web/packages/car/index.html.

[38] Lenth R, Singmann H, Love J, Buerkner P, Herve M. 2018 Emmeans: estimated marginal means, aka least-squares means. R package version 1, 3. See rdrr.io/cran/emmeans/man/emmeans.html.

[39] R Core Team. 2021 R: a language and environment for statistical computing. Vienna, Austria: R Foundation for Statistical Computing. See https://www.R-project.org/ (accessed on 2021).

[40] Wickham H. 2016 Ggplot2: elegant graphics for data analysis. Berlin, Germany: Springer.

[41] van Oppen MJH, Bongaerts P, Frade P, Peplow LM, Boyd SE, Nim HT, Bay LK. 2018 Adaptation to reef habitats through selection on the coral animal and its associated microbiome. Mol. Ecol. **27**, 2956-2971. (10.1111/mec.14763) 29900626

[42] Turnham KE, Wham DC, Sampayo E, LaJeunesse TC. 2021 Mutualistic microalgae co-diversify with reef corals that acquire symbionts during egg development. ISME J. **15**, 3271-3285. (10.1038/s41396-021-01007-8) 34012104 PMC8528872

[43] Sampayo EM, Franceschinis L, Hoegh-Guldberg O, Dove S. 2007 Niche partitioning of closely related symbiotic dinoflagellates. Mol. Ecol. **16**, 3721-3733. (10.1111/j.1365-294X.2007.03403.x) 17845444

[44] Ainsworth TD et al. 2021 Rebuilding relationships on coral reefs: coral bleaching knowledge-sharing to aid adaptation planning for reef users: bleaching emergence on reefs demonstrates the need to consider reef scale and accessibility when preparing for, and responding to, coral bleaching. Bioessays **43**, 2100048. (10.1002/bies.202100048) 34351637

[45] Sampayo EM, Ridgway T, Franceschinis L, Roff G, Hoegh-Guldberg O, Dove S. 2016 Coral symbioses under prolonged environmental change: living near tolerance range limits. Sci. Rep. **6**, 36271. (10.1038/srep36271) 27805069 PMC5090243

[46] Kaniewska P, Sampayo EM. 2022 Macro- and micro-scale adaptations allow distinct *Stylophora pistillata*-symbiodiniaceae holobionts to optimize performance across a broad light habitat. J. Phycol. **58**, 55-70. (10.1111/jpy.13215) 34612522

[47] Paz-García DA, Hellberg ME, García-de-León FJ, Balart EF. 2015 Switch between morphospecies of *Pocillopora* corals. Am. Nat. **186**, 434-440. (10.1086/682363) 26655359

[48] Mass T, Genin A. 2008 Environmental versus intrinsic determination of colony symmetry in the coral *Pocillopora verrucosa*. Mar. Ecol. Prog. Ser. **369**, 131-137. (10.3354/meps07578)

[49] Boron WF. 2004 Regulation of intracellular pH. Adv. Physiol. Educ. **28**, 160-179. (10.1152/advan.00045.2004) 15545345

[50] Barott KL, Barron ME, Tresguerres M. 2017 Identification of a molecular pH sensor in coral. Proc. R. Soc. B **284**, 20171769. (10.1098/rspb.2017.1769) PMC5698647 29093223

[51] Tresguerres M, Levin LR, Buck J. 2011 Intracellular cAMP signaling by soluble adenylyl cyclase. Kidney Int. **79**, 1277-1288. (10.1038/ki.2011.95) 21490586 PMC3105178

[52] Venn A, Tambutté E, Holcomb M, Allemand D, Tambutté S. 2011 Live tissue imaging shows reef corals elevate pH under their calcifying tissue relative to seawater. PLoS ONE **6**, e20013. (10.1371/journal.pone.0020013) 21637757 PMC3103511

[53] Barott KL, Venn AA, Thies AB, Tambutté S, Tresguerres M. 2020 Regulation of coral calcification by the acid-base sensing enzyme soluble adenylyl cyclase. Biochem. Biophys. Res. Commun. **525**, 576-580. (10.1016/j.bbrc.2020.02.115) 32115151

[54] Sevilgen DS, Venn AA, Hu MY, Tambutté E, Beer D, Planas-Bielsa V, Tambutté S. 2019 Full in vivo characterization of carbonate chemistry at the site of calcification in corals. Sci. Adv. **5**, eaau7447. (10.1126/sciadv.aau7447) 30746460 PMC6357752

[55] Venn AA, Tambutté E, Caminiti-Segonds N, Techer N, Allemand D, Tambutté S. 2019 Effects of light and darkness on pH regulation in three coral species exposed to seawater acidification. Sci. Rep. **9**, 2201. (10.1038/s41598-018-38168-0) 30778093 PMC6379376

[56] Zoccola D, Tambutté E, Kulhanek E, Puverel S, Scimeca J-C, Allemand D, Tambutté S. 2004 Molecular cloning and localization of a PMCA P-type calcium ATPase from the coral *Stylophora pistillata*. Biochim. Biophys. Acta **1663**, 117-126. (10.1016/j.bbamem.2004.02.010) 15157614

[57] Zoccola D et al. 2015 Bicarbonate transporters in corals point towards a key step in the evolution of cnidarian calcification. Sci. Rep. **5**, 9983. (10.1038/srep09983) 26040894 PMC4650655

[58] Barott KL, Perez SO, Linsmayer LB, Tresguerres M. 2015 Differential localization of ion transporters suggests distinct cellular mechanisms for calcification and photosynthesis between two coral species. Am. J. Physiol. Regul. Integr. Comp. Physiol. **309**, R235-R246. (10.1152/ajpregu.00052.2015) 26062631

[59] Tresguerres M, Barott KL, Barron ME, Deheyn DD, Kline DI, Linsmayer LB. 2017 Cell Biology of reef-building corals: ion transport, acid/base regulation, and energy metabolism. In Acid-base balance and nitrogen excretion in invertebrates (eds D Weihrauch, M O'Donnell), pp. 193-218. Berlin, Germany: Springer International Publishing.

[60] Vidal-Dupiol J et al. 2013 Genes related to ion-transport and energy production are upregulated in response to CO_2_-driven pH decrease in corals: new insights from transcriptome analysis. PLoS ONE **8**, e58652. (10.1371/journal.pone.0058652) 23544045 PMC3609761

[61] Mass T, Drake JL, Peters EC, Jiang W, Falkowski PG. 2014 Immunolocalization of skeletal matrix proteins in tissue and mineral of the coral *Stylophora pistillata*. Proc. Natl Acad. Sci. USA **111**, 12 728-12 733. (10.1073/pnas.1408621111) PMC4156710 25139990

[62] Barron ME, Thies AB, Espinoza JA, Barott KL, Hamdoun A, Tresguerres M. 2018 A vesicular Na^+^/Ca^2+^ exchanger in coral calcifying cells. PLoS ONE **13**, e0205367. 30379874 10.1371/journal.pone.0205367 PMC6209159

[63] Yuan X et al. 2018 Quantitative interpretation of vertical profiles of calcium and pH in the coral coelenteron. Mar. Chem. **204**, 62-69. (10.1016/j.marchem.2018.06.001)

[64] Cai W-J et al. 2016 Microelectrode characterization of coral daytime interior pH and carbonate chemistry. Nat. Commun. **7**, 11144. (10.1038/ncomms11144) 27041668 PMC4821998

[65] Brown KT, Mello-Athayde MA, Sampayo EM, Chai A, Dove S, Barott KL. 2022 Environmental memory gained from exposure to extreme pCO_2_ variability promotes coral cellular acid–base homeostasis. *Figshare*. (10.6084/m9.figshare.c.6168342) PMC9470260 36100023

